# Determination and Distribution of Polycyclic Aromatic Hydrocarbons in Rivers, Sediments and Wastewater Effluents in Vhembe District, South Africa

**DOI:** 10.3390/ijerph13040387

**Published:** 2016-03-30

**Authors:** Joshua N. Edokpayi, John O. Odiyo, Oluwaseun E. Popoola, Titus A. M. Msagati

**Affiliations:** 1 Department of Hydrology and Water Resources, University of Venda, Private Bag X5050, Thohoyandou 0950, South Africa; john.odiyo@univen.ac.za; 2 Department of Chemical Sciences, Yaba College of Technology, P.M.B. 2011 Yaba, Lagos 101212, Nigeria; seunliz27@yahoo.com; 3 College of Science, Engineering and Technology, Nanotechnology and Water Sustainability Research Unit, Florida Science Campus, University of South Africa, 1710 Roodepoort, Johannesburg 2000, South Africa; msagatam@unisa.ac.za

**Keywords:** contamination, polycyclic aromatic hydrocarbons, river, sediments, wastewater

## Abstract

Polycyclic aromatic hydrocarbons are very toxic and persistent environmental contaminants. This study was undertaken to assess the concentrations and possible sources of 16 PAHs (Polycyclic aromatic hydrocarbons) classified by the United State Environmental Protection Agency as priority pollutants in water and sediments of the Mvudi and Nzhelele Rivers. Effluents from Thohoyandou wastewater treatment plant and Siloam waste stabilization ponds were also investigated. Diagnostic ratios were used to evaluate the possible sources of PAHs. PAHs in the water samples were extracted using 1:1 dichloromethane and *n-hexane* mixtures, while those in the sediment samples were extracted with 1:1 acetone and dichloromethane using an ultrasonication method. The extracts were purified using an SPE technique and reconstituted in *n-hexane* before analyses with a gas chromatograph time of flight—mass spectrometer. The results obtained indicate the prevalence of high molecular weight PAHs in all the samples. PAHs concentrations in water and sediment samples from all the sampling sites were in the range of 13.174–26.382 mg/L and 27.10–55.93 mg/kg, respectively. Combustion of biomass was identified as the major possible source of PAHs. Effluents from wastewater treatment facilities were also considered as major anthropogenic contributions to the levels of PAHs found in both river water and sediments. Mvudi and Nzhelele Rivers show moderate to high contamination level of PAHs.

## 1. Introduction

Polycyclic aromatic hydrocarbons (PAHs) are ubiquitous in the environment and are part of numerous organic contaminants that are persistent in the environment, have long transport potential and can cause adverse environmental effects [1,2]. Some of them are susceptible to dispersion on a global scale because, in addition to having environmental persistence, they are “semi-volatile”, *i.e.,* under environmental conditions they move between the atmosphere and the earth’s surface in repeated, temperature-driven cycles of deposition and volatilisation [3,4]. PAHs are truly multimedia contaminants which occur in all parts of the environment: atmosphere, inland and sea waters, sediments, soils and vegetation [2,3]. 

There are thousands of PAH compounds in the environment but in practice PAH analysis is restricted to a few compounds—mostly the 16 priority compounds (Naphthalene, Acenaphthylene, Acenaphthene, Fluorene, Phenanthrene, Anthracene, Fluoranthene, Pyrene, Benz[a]anthracene, Chrysene, Benzo[b]fluoranthene, Benzo[k]fluoranthene, Benzo[a]pyrene, Indeno[1,2,3-cd]pyrene, Dibenzo[a,h]anthracene and Benzo[ghi]perylene) listed by US EPA as potentially toxic [5]. Individual PAHs differ substantially in their physical and chemical properties [6,7]. Generally, PAHs are hydrophobic with very little solubility in water which decreases with increasing molecular weight or the number of fused aromatic rings. The high molecular weight (HMW) PAHs (≥ 4 fused aromatic rings) are less water-soluble, less volatile and more lipophilic than lower molecular weight (LMW) PAHs (≤3 fused aromatic rings) [2,3,8]. PAHs are not part of the 12 priority pollutants of the Stockholm convention but are listed by the United States Environmental Protection Agency and the European Commission as priority pollutants [9,10,11]. The widespread occurrence of PAHs is largely due to their formation and release in all processes of incomplete combustion of organic materials. The last century of industrial development caused a significant increase of PAH concentrations in the natural environment [2,8].

Natural sources of pyrogenic PAH such as volcanic activity and forest fires do not significantly contribute to overall PAH emission [12,13]. These pollutants are mostly formed during the incomplete combustion and pyrolysis of fossil fuels or wood and from the release of petroleum products [7,14]. Other sources of PAHs include petroleum spills, oil seepage and diagenesis of organic matter in anoxic sediments [2,15]. PAHs are also found in coal tar, crude oil, creosote and roofing tar and a few are used in medicine or to make dyes, plastics, and pesticides [6,16,17]. PAHs have also some beneficial applications as some of them are produced for commercial use; these include naphthalene, fluorene, anthracene, phenanthrene, fluoranthene, and pyrene [18]. Exposure to PAHs causes a variety of negative health impacts which includes: reproductive defects, DNA mutations, leukemia and cancer of the lung, bladder, bone, brain and scrotal [19,20]. Several water and sediments quality guidelines for the assessment of ecological risk of PAHs on benthic and other aquatic organisms are widely reported in the literature [21,22,23,24]. The effect range low (ERL) and effect range medium (ERM) based approaches formulated by Long and MacDonald [25] have been widely applied [26]. 

Although PAHs have received much attention in terms of scientific research in some developed countries because of the adverse effects they have on the environment, animals and human health, major data gaps exist on its sources and levels in developing countries [12,27,28]. South Africa is a leading industrialized nation in the African continent but it has not put much emphasis on the monitoring of the levels of PAHs in its environment [27,28]. There is also limited knowledge on the extent to which burning of waste/forest contributes to the PAHs emissions in South Africa [27,28,29]. The contribution of wastewater as potential sources of PAHs in sediments and soil when used for irrigation in South Africa is also lacking. This study was conducted to determine the levels and distribution of PAHs in Surface water and sediments including wastewater effluents in Vhembe District of South Africa.

**Figure 1:**
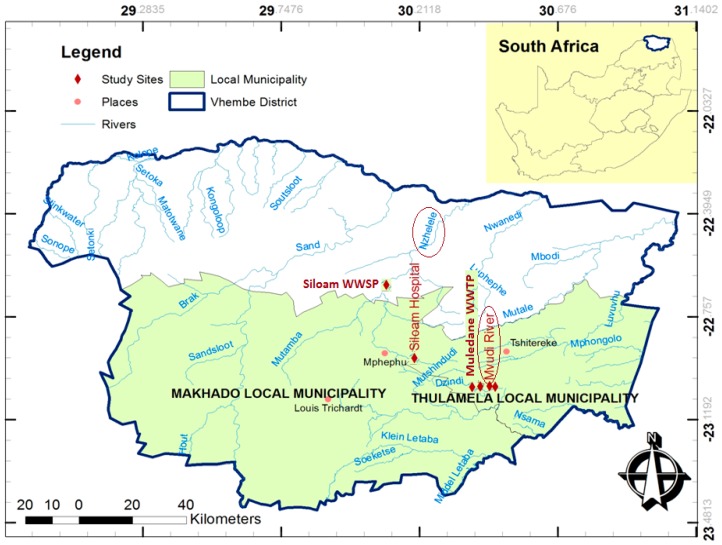
Map of Vhembe District.

The study area is located in Vhembe District which is situated between longitudes 28°54′20.66′′ E and 31°04′31.19′′ E and latitudes 22°7′26.59′′ S and 23°26′6.49′′ S in the northern region of Limpopo Province of South Africa (Figure 1). The study area is made up of various urban and semi-urban settlements as well as numerous scattered rural villages. Land is predominantly used for commercial/subsistence agriculture and rural settlements. Solid waste disposal sites and wastewater treatment facilities also make up part of land use. Furthermore, the study area is characterised by fired brick-making, pottery, paint industries and wood creosote (rich in PAHs) treatment plants. Burning of lands in preparation for agriculture, refuse and burning of vehicle tyres are common features in the study area. This region is characterised by a warm wet season which is associated with high temperatures up to 40 °C and is usually between October and March, with peak precipitation in January and February. The cool dry season has a temperature range between 12 and 22 °C and begins from April to September [30].

## 2. Materials and Methods 

### 2.1. Chemicals

Acetone (99.8%), dichloromethane (99.5%), *n-hexane* (99.8%) and acetonitrile were purchased from Merck (pty) Ltd (Johannesburg, South Africa). Sodium chloride and anhydrous sodium sulphate were obtained from Fluka (Steinheim, Germany). Solid phase extraction (SPE) C18 cartridges and certified reference materials of 16 USEPA PAHS were purchased from Sigma-Aldrich (St. Louis, MO, USA). All reagents were used without any further purification. 

### 2.2. Sampling

Water and sediment samples were collected from three different points in Mvudi (30°28′28′′ E and 23°0′13′′ S) and Nzhelele (30°22′19′′ E and 22°2′08′′ S) Rivers. Wastewater effluent samples were also collected from Thohoyandou wastewater treatment plant (WWTP) (30°28′48′′ E and 23°0′00′′ S) and Siloam wastewater stabilisation ponds (WSPs) (30°11′10′′ E and 30°11′23′′ S). The samples were transported on ice chest to Hydrology and Water Resources laboratory of the University of Venda. The water samples were kept at 4 °C in EPA vials with Teflon crew cap as recommended for PAH samples before analysis [31]. The sediment samples were placed into polyethylene plastic bags and transported in a similar fashion as the water samples. In the laboratory, sediment samples were air dried, homogenized, ground gently with an agate pestle and mortar and sieved with a 500 µm analytical sieves. 

### 2.3. Extraction and Analyses of PAHs

Liquid-liquid extraction was used for the extraction of PAHs in water samples. EPA [31] method and slightly modified procedure reported by Nekhavhambe *et al.* [32] was employed. 75 g of NaCl was added to 500 mL of the sample in a separating funnel. PAHs in the sample were extracted three times using a mixture of dichloromethane and *n-hexane*. After vigorous shaking, the extract was dehydrated using anhydrous sodium sulfate, and concentrated to 2.0 mL with a rotary evaporator at 35–40 °C. Organic contaminants in extracts were purified using SPE florisil columns, and was concentrated and reconstituted using 1 mL of *n-hexane* and analysed using gas chromatography time of flight mass spectrometer (GC-TOF-MS). The extraction of sediment samples was performed using the method outlined by Oluseyi *et al.* [33] where 1 g of each sediment sample was weighed into pre-cleaned 25 mL amber glass bottles. 10 mL of 1:1 acetone:dichloromethane were added, respectively. The bottles were sealed with screw cap closure lined with a PTFE-faced silicone rubber septum and shaken vigorously to suspend the contents. The bottles were then sonicated in a high performance ultrasonic bath with microprocessor control for precision time and temperature controlled operation for 60 min at 50 °C. The sample bottles were intermittently inverted and shaken to continually re-suspend the samples. SPE clean-up of the sediment extracts was carried out with 6 mL Supelco C18 SPE cartridges. The extraction solutions were each loaded and aspirated through the cartridge under gentle vacuum at a flow rate of less than 2 mL/min. The collected extract was reconstituted in 1 mL *n-hexane* and analysed using GC-TOF-MS.

### 2.4. Validation Studies and Preparation of Calibration Standards

Spiked recovery method was used for validation studies for both the water and sediment samples. The samples were spiked with 1 µL of 100 mg/L standard mixture consisting of 16 PAHs to 500 mL pre-extracted water samples. Double-distilled water (500 mL) was first pre-extracted in triplicate with 30 mL dichloromethane as a blank sample. The spiked samples were then extracted and analysed. PAHs standards were used for the calibration of the instrument. The calibration standards were prepared by serial dilution from stock solution. 

### 2.5. Instrument and Analytical Conditions

Analyses of PAHs were performed using gas chromatograph (Model 7890 series, Agilent Technologies Inc., Wilmington, DE, USA) coupled to a LECO Pegasus 4D Time of Flight mass spectrometer (St. George, MI, USA). The column set comprised of a primary column, Rxi-5SilMS (30 m × 0.25 mm internal diameter, 0.25 µm stationary film thickness) and a secondary column Rxi-200 (1 m × 0.15 mm internal diameter, 0.15 µm stationary film thickness). Helium was used as the carrier gas whereas nitrogen, compressed air and liquid nitrogen were used for the operation of the quad jet thermal modulator. The sample injector temperature was set at 250 °C and samples were injected at a volume of 1 µL in splitless mode. The flow of carrier gas was set at a rate of 1.5 mL/min. The oven temperature was programmed as follows: 80 °C held for 0.5 min; ramped from 80 to 220 °C at 20 °C/min, then 220–300 °C at 10 °C/min. The mass spectrometry conditions were set as follows: Ionisation: electron ionisation at −70 eV; source temperature: 180 °C; stored mass range: 47–350 µm; acquisition rate: 20 spectra/second; detector voltage: −1500 V.

## 3. Results

### 3.1. Calibration and Percentage Recovery

The calibration graph obtained by plotting the peak areas against the concentration of the analyte from the certified reference materials were all linear with correlation coefficients ranging between 0.971 and 0.999. Acceptable recoveries in the range of 95.9%–149% were obtained. 

### 3.2. PAH Concentrations

PAHs do not usually exist as separate entities in environmental media; they are often regarded as a mixture and the total concentration of their mixture is often used to describe their distribution. 16 PAHs recommended by the United State Environmental Protection Agencies (US EPA) were investigated in this study but only 10 of them were determined in all of the sampling sites during the course of this study (Table 1) while the other 6 were below detectable limits. The concentrations of PAHs in Mvudi and Nzhelele River water varied between 0.126 and 7.510 mg/L and below detection limits to 7.805 mg/L, respectively. Mvudi River water (16.585 mg/L) recorded higher total PAH levels than Nzhelele River water (15.134 mg/L). Similarly, the levels of PAHs determined in Mvudi and Nzhelele River sediments range between 0.266 and 21.60 mg/kg and 0.206 and 13.71 mg/kg, respectively. The total PAHs in Mvudi River sediment (55.93 mg/kg) was also higher than Nzhelele River sediment (27.10 mg/kg). The levels of PAHs in Thohoyandou WWTP and Siloam WSPs ranges from below detection limit to 7.510 mg/L and 8.310 mg/L, respectively. The effluents from Siloam WSPs recorded higher total PAHs levels (26.38 mg/L) than Thohoyandou WWTP (13.17 mg/L) (Table 1). This reasoning can be supported by another study by Qi *et al.* [34] who reported total PAHs in the range of 0.245–0.4040 mg/L and 0.43–2.860 mg/L in wastewater effluent and wastewater from small sewer in Beijing, China. Both sources were regarded as contributors of PAH loadings into river sources. Therefore, both wastewater treatment facilities in this study can be regarded as point sources of PAHs contamination to the rivers.

Generally, the lower molecular weight (LMW) PAHs were present in lower concentrations in all sampling sites except for fluorene. Fluoranthene and benzo[b]fluoranthene were present in higher concentration when compared to other PAHs in all sampling sites. Nekhavhambe *et al.* [32] reported the levels of PAHs in some rivers and sediments in Vhembe District of South Africa. They reported a mean value of 0.1092 mg/L and 0.056 mg/L for Mutshundudi and Nzhelele Rivers waters and 11.711 mg/kg and 7.035 mg/kg for both rivers sediments, respectively. Their results showed lower total PAH concentrations in the Mutshundudi and Nzhelele Rivers and their sediments when compared to the results of this study (Table 1). PAHs are known for their persistence in the environment and more PAHs could have accumulated within 8 years, thus the higher concentration in this study. This is similar to reasons for the findings of Elder and Dresler [35] on the accumulation and bio-concentration of PAHs in estuarine environment in Florida, USA. Also, only 6 PAHs were used for computing the total PAH concentrations in the water and sediments of those rivers whereas 10 PAHs were considered in this study (Table 1).

Generally, the concentrations of PAHs were higher in the sediments of both river than in the water samples. This is due to the hydrophobic nature of PAHs [36]. PAHs tends to adsorb on the surface of sediments because they are not soluble in water. The results of this study showed higher levels of PAHs than those reported by Sibiya *et al.* [37] on the application of solid phase extraction (SPE) method for polycyclic aromatic hydrocarbons in water samples in Johannesburg area, South Africa; also, 5 PAHs were used for computing the total PAHs in their study. 

Correlation analysis reveals that only naphthalene (*r* = 0.974, *p* < 0.05) and benzo[b]fluoranthene (*r* = 0.965, *p* < 0.05) correlated positively and significantly in the water and sediments of Mvudi River, acenapthene (*r* = −1.00, *p* < 0.01), pyrene (*r* = −1.00, *p* < 0.01) and fluoranthene (*r* = −0.99, *p* < 0.01), correlated negatively but significantly of the same river. Other PAHs did not show any significant correlation. The mean difference of levels of each of the PAHs in water and sediments of Mvudi River varied significantly for all PAHs determined except acenapthene and acenapthylene. Similarly, in the water and sediments of Nzhelele River, acenathylene (*r* = 0.971, *p* < 0.05), pyrene (*r* = 0.999, *p* < 0.01), flouranthene (*r* = 0.968, *p* < 0.01) and benzo[b]fluoranthene (*r* = 0.995, *p* < 0.01) correlated positively and significantly. Phenanthrene (*r* = −1.00, *p* < 0.01), acenapthene (*r* = −1.00, *p* < 0.01) and benzo[a]pyrene (*r* = −0.999, *p* < 0.01) correlated negatively but significantly. The mean difference between the levels of each of the PAHs in water and sediments of Nzhelele River varied significantly for all PAHs except for acenapthene and benzo[a]pyrene. Anthracene was not computed because it was found below the detection limit of the analytical instrument. 

### 3.3. Sources of PAHs

The sources of PAHs can either be petrogenic *i.e.,* released from petroleum products or pyrogenic due to the combustion of biomass. Diagnostic ratios have been designed and used to distinguish the sources of PAHs due to their stability, physical and chemical attributes [38,39]. The most commonly used ratios for the source identification of PAHs are presented in Table 1 and Table 2. 

Table 1 shows the diagnostic ratios of the PAHs obtained in this study and their possible sources. The ratio of Anthracene/(Anthracene+Phenanthrene), in Mvudi and Nzhelele Rivers and sediments were >0.1 except for Nzhelele River water which implies that the source of the PAHs was from pyrogenic sources due to the combustion of bushes and other biomass [39]. This ratio was not applied to the effluents from Thohoyandou WWTP and Siloam WSPs as anthracene was not detected in their samples. 

The ratio (Low Molecular weight PAHs)/(High Molecular weight PAHs) gave values of <1 for all the sampling sites supporting the fact that the source of the PAHs was more likely to be due to the combustion of biomass. Also, the use of Fluoranthene/(Fluoranthene+Pyrene) ratio gave values of >0.5, which further comfirms a pyrogenic source. Otherwise, values of <0.4 would have implied that the source of the PAHs was due to the use of petroleum products. Burning of farmland for agriculture and refuse is a common practice around Thohoyandou. Generally, the source of PAHs in both river systems and the wastewater treatment facilities (WWTFs) can be attributed to pyrogenic activities although petrogenic contribution cannot be ruled out. Most PAHs that are persistent in environmental media are usually from pyrogenic sources rather than petrogenic sources.

### 3.4. PAH Compositions

**Figure 2:**
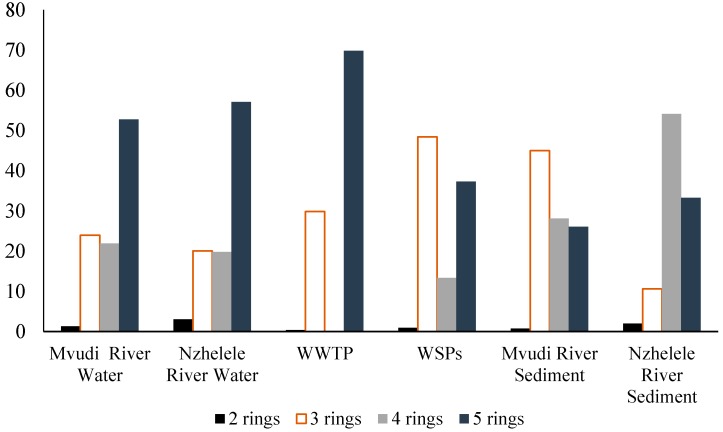
Percentage PAHs composition in the various sampling points.

**Figure 3:**
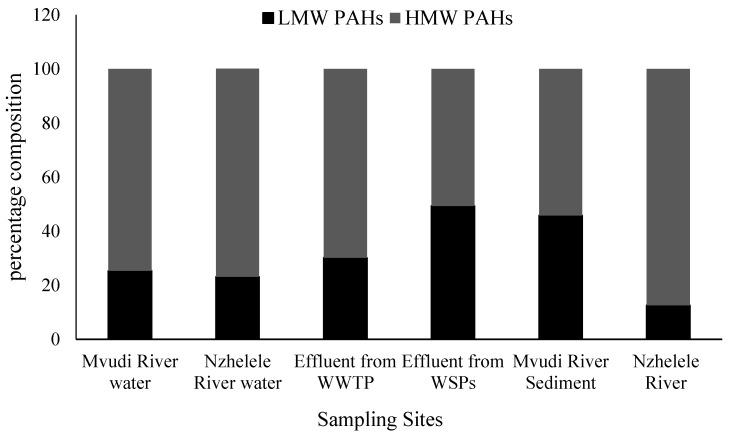
Percentage composition of LMW and HMW PAHs.

The composition pattern of PAHs detected in the samples by the number of rings are shown in Figure 2. Generally, the high molecular weight (HMW) PAHs with ≥4 rings was predominant in the rivers, sediments and WWTFs samples making up to 74.7%, 76.9%, 69.83%, 50.7%, 54% and 87% in Mvudi River, Nzhelele River, Thohoyandou WWTP, Silaom WSPs, Mvudi sediments and Nzhelele sediments, respectively (Figure 3). This may be attributed to their low solubility in water, less volatility due to their molecular size and higher persistence in aqueous environment when compared to the low molecular weight (LMW) PAHs [41]. The major source of HMW PAHs can be linked to anthropogenic activities [42]. 

HMW PAHs are more persistent than LMW PAHs in the environment due to their increased resistance to oxidation, reduction and vapourisation as molecular weight increases [43]. LMW PAHs such as naphthalene and fluorene have more significant acute toxicity to aquatic organisms than HMW PAHs but are non-carcinogenic. Some HMW PAHs such as benzo[a]pyrene and benzo[b] fluoranthene are carcinogenic and mutagenic to a wide variety of organisms including fish, birds and mammals [44]. Five molecular weight PAHs were more prevalent in the water of Mvudi and Nzhelele Rivers and effluent of Thohoyandou WWTP. Four ring PAHs were only prevalent in the sediments of Nzhelele River. Siloam WSPs and the sediments of Mvudi River contained more of three ring PAHs than others. Two ring PAHs were the least determined in all the sampling sites. Generally, HMW PAHs were higher in all the sampling sites than LMW PAHs. 

The physical and chemical characteristics of PAHs vary in accordance to their number of rings. The greater the number of rings is, the higher the molecular weight. As the molecular weight increases, the solubility of PAHs decreases with an increase in melting and boiling point. The number of rings in PAHs also determine their toxicity [45]. Nasher *et al.* [39] reported the prevalence of HMW PAHs in water collected around Langkawi Island in Malaysia. They attributed their findings to anthropogenic activities such as incomplete fuel combustion and vehicle engine emissions. Zhao *et al.* [46] and Okedeyi *et al.* [29] also reported the prevalence of HMW PAHs in their studies around Mai Po inner deep bay of Hong Kong and coal fired power plants in South Africa, respectively. The comparisons of Total PAH obtained in this study and different parts of the world is presented in Table 3.

### 3.5. Health Risk Assessment

The three major routes of exposure of PAHs to humans are inhalation, ingestion and dermal contact [43,45]. The potential toxicity of PAHs in water and sediments can be calculated in relation to benzo[a]pyrene, which is the most carcinogenic PAH. The potential toxicity can be calculated using the relation reported by Nekhavhambe *et al.* [32] in Equation (1):

(1) $$\mathrm{Total}\mathrm{TEQ}=\sum_{i}C_{i}\times TEF_{i}$$

where TEQ = toxic equivalent quotient, *C_i_* =concentration of individual PAHs, *TEF_i_* = toxic equivalent factor relative to benzo[a]pyrene. The values obtained are presented in Table 4. The computed TEQ values were higher in the sediment samples than the river water and effluent samples. The TEQ values of the effluents from WWTFs are high; with respect to benzo[a]pyrene, workers in the wastewater treatment facilities must be protected from direct contact with the wastewater in order to reduce the likelihood of skin cancer [54]. 

The potential toxicity of PAHs in the sediments on the surrounding aquatic organisms and their ecosystem was also assessed. PAHs levels in the sediments were compared with the US National Oceanic sediment quality guidelines [23]. Table 4 shows the recommended ERL (effect range low) and ERM (effect range median) target values. Values above the recommended ERM values indicate the likelihood of occurrence of high negative toxic effect in that area. Mild toxic effect is expected if the PAHs concentrations range between ERL and ERM values [25]. No negative effect is expected for PAH concentrations lower than ERL values. From the results presented in Table 5, there is a high probability of risk for the organisms that live in both Mvudi and Nzhelele Rivers. Anthracene, naphthalene and phenanthrene exceeded the ERL values but fell within the ERM values for both rivers, suggesting a mild negative toxic effects on aquatic organisms present in the rivers. Pyrene exceeded the ERL value in Nzhelele River sediments but fell within the ERM value; conversely, pyrene was below the ERL value in Mvudi River. Acenaphthene, acenaphthylene and fluoranthene exceeded ERM values for both rivers. Benzo[a]pyrene and flourene only exceeded the ERM values for the Mvudi River. Based on the results of this study, occasionally adverse biological effects (such as cancer and reproductive and physiological disorders) may occur in fish, birds and mammals [56]. 

## 4. Conclusion

The distribution of PAHs in the samples indicates a possible health risk to humans and aquatic organisms as some of the individual PAHs exceeded the ERM recommended values. Mvudi River sediment was the most contaminated site investigated. LMW PAHs contributed little to the total PAH concentration in all the sampling sites. Sediment samples were more contaminated than the water samples. WWTFs in Vhembe District are regarded as potential sources of PAHs in both river waters and sediments. Results from diagnostic ratios favour a pyrogenic source of PAH pollution over a petrogenic source, although the contribution of the latter cannot be ignored. 

## Figures and Tables

**Table 1:** PAHs concentration in the sampling sites.

PAHs (mg/L or mg/kg)	Rings	Mvudi River Water	Nzhelele River Water	WWTP	WSPs	Mvudi River Sediment	Nzhelele River Sediment
Naphthalene	2	0.222	0.458	0.047	0.254	0.500	0.206
Anthracene	3	0.256	Bdl	Bdl	Bdl	0.440	0.540
Fluorene	3	2.480	0.002	2.200	6.270	21.60	0.311
Phenanthrene	3	0.126	0.177	1.728	6.495	0.778	0.206
Acenaphthene	2	0.579	0.423	Bdl	Bdl	0.738	1.135
Acenaphthylene	2	0.537	0.765	Bdl	Bdl	1.541	0.725
Pyrene	4	1.138	0.573	Bdl	1.186	0.266	0.961
Fluoranthene	4	2.498	2.423	Bdl	2.340	15.47	13.71
Benzo[b]fluoranthene	5	7.510	7.805	7.510	8.310	10.74	7.970
Benzo[a]pyrene	5	1.239	0.840	1.659	1.447	3.859	1.050
LMW PAHs		4.200	3.493	3.975	13.02	25.60	3.410
HMW PAHs		12.39	11.64	9.199	13.36	30.93	23.69
Total PAHs		16.59	15.13	13.17	26.38	55.93	27.10
LMW/HMW		0.340	0.300	0.600	0.970	0.840	0.140
Flu/Flu+Py		0.690	0.930	-	0.670	0.980	0.930
Ant/Ant+Phen		0.670	0.070	-	-	0.360	0.520

Flu is fluoranthene, Py is pyrene, Ant is anthracene, phen is phenanthrene and Bdl is below detection limit.

**Table 2:** Characteristic diagnostic ratios values for particular pollution emission sources.

Diagnostic Ratio	Petrogenic	Pyrogenic	References
Ant/Ant+Phen	<0.1	>0.1	Brandli *et al.* [40]
Flu/Flu+Py	<0.4	>0.4	Brandli *et al.* [40]
LMW/HMW	>1	<1	Nasher *et al.* [39]
	Fuel combustion	Grass/coal/wood combustion	

Ant is anthracene, Phen is phenanthrene, Flu is fluoranthene and Py is pyrene.

**Table 3:** Total concentrations of PAH in river sediments from different locations in the world.

Location	Total PAHs (ng·g^−1^)	Number of PAH Compounds	Reference
Klang River, Malaysia	3803.2–7442.7	16	Keshavarifard *et al.* [1]
Donggang River, Taiwan	23–2534	16	Hsieh *et al.* [47]
Evrotas River, Greece	0.3–195.4	8	Tzoraki *et al.* [48]
Warri River, Nigeria	42–2298.7	11	Asagbra *et al.* [49]
Negro River, Brazil	5.6–1187	16	Souza *et al.* [50]
Haihe River, China	774.81–255,371.91	16	Jiang *et al.* [51]
Arc River, France	153–1311	17	Kanzari *et al.* [52]
Gomti River, India	5.24–3722.87	16	Malik *et al.* [53]
Nzhelele River, South Africa	206–13,710	10	This study
Mvudi River, South Africa	440–21,600	10	This study

**Table 4:** TEF and TEQ values of PAHs.

PAHs	TEF Values	Mvudi River Water	Nzhelele River Water	WWTP	WSPs	Mvudi River Sediment	Nzhelele River Sediment
Naphthalene	0.001 ^b^	0.000222	0.000458	0.000047	0.000254	0.0005	0.000204
Anthracene	0.01 ^b^	0.00256	Bdl	Bdl	Bdl	0.0044	0.0054
Fluorene	0.001 ^b^	0.00248	0.00167	0.0022	0.00627	0.0216	0.000311
Phenanthrene	0.001 ^b^	0.0001258	0.000177	0.001728	0.0065	0.000778	0.000497
Acenaphthene	0.001 ^b^	0.000579	0.000423	Bdl	Bdl	0.000738	0.001135
Acenaphthylene	0.001 ^b^	0.0005368	0.000765	Bdl	Bdl	0.00154	0.000725
Pyrene	0.001 ^a^	0.001138	0.000573	Bdl	0.001186	0.000266	0.000961
Fluoranthene	0.08 ^a^	0.19984	0.19384	Bdl	0.1872	12.376	10.968
Benzo[b]fluoranthene	0.8 ^a^	6.008	6.244	6.032	6.712	8.592	6.376
Benzo[a]pyrene	1 ^a^	1.239	0.84	1.659	1.447	3.859	1.05
∑TEQ		7.4543	7.282	7.6950	8.3604	24.86	18.40

^a^ = US EPA [55], ^b^ = Nekhavhambe *et al.* [32]. Bdl is below detection limit.

**Table 5:** Concentrations of PAHs in Mvudi and Nzhelele Rivers sediment and toxicity guidelines.

PAHs	ERL (ng·g^−1^)	ERM (ng·g^−1^)	Mvudi Sediment (ng·g^−1^)	Nzhelele Sediment (ng·g^−1^)
Naphthalene	160	2100	500	206
Anthracene	85	1100	440	540
Fluorene	19	540	21,600	311
Phenanthrene	240	1500	778	497
Acenaphthene	16	500	738	1135
Acenaphthylene	44	640	1541	725
Pyrene	665	2600	266	961
Fluoranthene	600	5100	15,470	13,710
Benzo[b]fluoranthene	-	-	-	-
Benzo[a]pyrene	430	2800	3859	1050

## Abbreviations

The following abbreviations are used in this manuscript:

WWTP	Wastewater treatment plant
WSPs	Waste stabilization ponds
WWTFs	Wastewater treatment facilities
ERL	Effect range low
ERM	Effect range medium
PAHs	Polycyclic aromatic hydrocarbons
DWAF	Department of Water Affairs and Forestry
WHO	World Health Organization
US EPA	United States Environmental Protection Agency
